# 232. Malnutrition is Associated with Poor Outcomes in Patients Hospitalized with *Staphylococcus aureus* Bacteremia

**DOI:** 10.1093/ofid/ofad500.305

**Published:** 2023-11-27

**Authors:** Baraa Saad, Nourhan Saleh, Aseel Alkhader, Nikita Garg, David Reynoso

**Affiliations:** UTMB, League city, Texas; Alexandria University, Alexandria, Al Iskandariyah, Egypt; Jordan University of Science and Technology, Irbid, Irbid, Jordan; UTMB, League city, Texas; The University of Texas Medical Branch, Galveston, Texas

## Abstract

**Background:**

*Staphylococcus aureus* bacteremia (SAB) is one of the most common and clinically significant bacterial infections worldwide. Malnutrition has been linked to poor outcomes in various infectious and non-infectious diseases. The objective of this study is to investigate the impact of malnutrition on the outcomes among adult patients hospitalized with SAB.

**Methods:**

A cohort study was conducted using the Nationwide Inpatient Sample (2016-2019). Adult hospitalizations with Methicillin-Sensitive or -Resistant *Staphylococcus aureus* bacteremia were identified and stratified by the presence of malnutrition using ICD-10 codes. The primary outcome was in-hospital mortality. Secondary outcomes included ICU admissions, septic shock, mechanical ventilation, length of stay (LOS), and hospital charges. Multivariate regression analysis adjusted for confounding factors.

**Results:**

Of 518,840 SAB patients, 14.10% had malnutrition. Malnourished patients showed higher rates of mortality, septic shock, mechanical ventilation, mean LOS, and hospital charges. Multivariate analysis revealed malnutrition as an independent predictor of worse outcomes. Adjusted odds ratios were 1.60 for mortality (P< 0.001), 1.69 for septic shock (P< 0.001), and 1.75 for mechanical ventilation (P< 0.001). Adjusted mean LOS and total charges increased by 6.14 days and $87,885 for malnourished SAB patients. Outcomes were similar for MSSA and MRSA.

**Figure:**
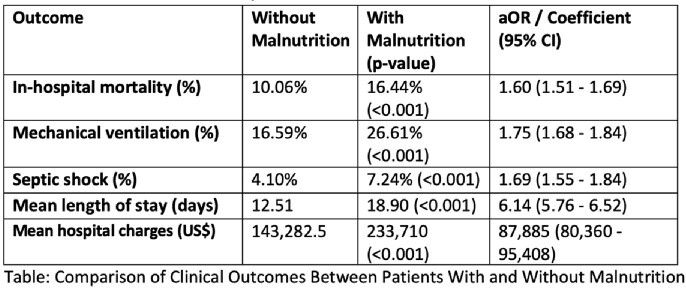

**Conclusion:**

Our study highlights the significant impact of malnutrition on outcome among patients hospitalized with SAB. We found that malnourished individuals with SAB had higher rates of mortality, septic shock, mechanical ventilation, prolonged hospital stays and increased financial burden. These results emphasize the critical need to address and recognize malnutrition as a significant risk factor in the management of SAB. Further research and interventions targeting the issue of malnutrition in SAB patients are needed to enhance outcomes, decrease associated mortality and morbidity, and reduce healthcare cost.

**Disclosures:**

**All Authors**: No reported disclosures

